# Risk factors for 90-day mortality after pancreaticoduodenectomy

**DOI:** 10.1186/s12893-025-03461-z

**Published:** 2025-12-24

**Authors:** Mesut Yur, Erhan Aygen, Yavuz Selim İlhan, Cüneyt Kırkıl, Serkan Yılmaz, Ahmet Akbaş

**Affiliations:** 1 Department of Surgical Oncology, Fırat University, Elazığ, Turkey; 2 Department of General Surgery, Fırat University, Elazığ, Turkey; 3 Department of General Surgery, Karadeniz Technical University, Trabzon, Turkey

**Keywords:** Pancreaticoduodenectomy, Mortality, MELD-Na, Risk factors

## Abstract

**Background:**

Pancreaticoduodenectomy (PD) is a key surgical procedure for periampullary cancer, but it has a concerning postoperative mortality rate. This study evaluates the risk factors influencing 90-day mortality after PD in periampullary cancer patients.

**Methods:**

Between January 2010 and August 2022, 105 eligible patients with PD were included in the study. Demographic, pathological, and laboratory data were collected for this research. The study also included the MELD-Na score, the age-adjusted Charlson Comorbidity Index (ACCI), and the neutrophil-lymphocyte ratio (NLR). The patients were divided into two groups: those who experienced 90-day mortality (17 patients in group I) and those who were discharged (88 patients in group II). Kaplan-Meier analysis and Cox proportional hazards model were utilized to identify the prognostic factors associated with 90-day mortality.

**Results:**

In the univariate analysis, age, sex, albumin levels, NLR, MELD-Na scores, and ACCI were significantly associated with 90-day postoperative mortality (p˂0.05). In multivariate analysis, the MELD-Na score at the admission (HR: 10.75; 95% CI: [2.82–61.30]; *p* = 0.001), ACCI (HR: 9.30; 95% CI: [2.30-46.05]; *p* < 0.001), and NLR (HR: 7.81; 95% CI: [1.98–48.38]; *p* = 0.005) were independent risk factors for postoperative 90-day mortality.

**Conclusions:**

Although several factors affect 90-day postoperative mortality after pancreaticoduodenectomy, the MELD-Na score, ACCI, and NLR are independent risk factors. Assessing patients in the risk group separately by a multidisciplinary team may reduce mortality.

## Background

Cancers in the ampulla of Vater, distal common bile duct, pancreatic head, and the second part of the duodenum are known as periampullary cancers (PAC). The most common reason for hospital referral for PAC is painless jaundice secondary to biliary obstruction [1]. Hyperbilirubinemia and bile acid stasis occur due to this obstruction. This condition adversely affects numerous systems, including the liver, kidneys, intestines, immune system, hemostasis, and wound healing [2]. The tumors are mostly unresectable or present with distant metastases at diagnosis. Pancreaticoduodenectomy (PD) is the most commonly performed surgical technique for patients with resectable tumors and prolongs overall survival (OS) [3]. Dr. Allen O. Whipple performed the first successful PD procedure in 1934 [4]. Perioperative mortality, which was initially high, has decreased over time and is now nearly 0%. However, early postoperative mortality remains at 6%, even in high-volume hospitals, and is still a critical issue for patients and physicians [5, 6]. Moreover, the mortality rate could increase to 20% due to the low volume of hospitals and surgeons’ experience [7–10].

The Model for End-Stage Liver Disease (MELD) scoring system was created to estimate early mortality in patients with chronic liver disease [11]. It is calculated based on creatinine, INR, and bilirubin levels. It was initially used in patients undergoing transjugular intrahepatic portosystemic shunt surgery. Subsequently, it was used as a priority and prognostic marker for patients undergoing transplants [12, 13]. Then, the MELD-Na score was used by adding Na^+^ to the MELD score. The MELD-Na score estimates the 90-day mortality rate in patients with chronic liver disease. Studies have also been conducted to predict early postoperative mortality in chronic liver disease and liver malignancies [12, 14–16]. The association between MELD-Na scores and early mortality (90-day mortality) in patients undergoing PD due to PAC has not been studied before.

The neutrophil-lymphocyte ratio (NLR) is another parameter investigated for early mortality prediction. NLR is quickly and inexpensively obtained through blood counts and is calculated by dividing the neutrophil count by the lymphocyte count. Previous studies have shown that NLR has a prognostic effect on early mortality after gastrointestinal system surgery in chronic liver disease patients with extra diseases such as heart disease, pancreatitis, and sepsis [17–22]. A study of NLR for 90-day mortality after PD in PACs (regardless of tumor location) has not yet been performed.

Previously, MELD-Na and NLR have been investigated in many diseases and have been helpful in predicting early mortality [12, 14, 20, 22]. We believe these parameters may also provide beneficial results for patients undergoing PD for PAC. The aim of this study was to reinvestigate the parameters affecting 90-day mortality with other parameters not previously studied.

## Methods

### Ethical approval and patient selection

The study was initiated after receiving approval from the ethics committee (Noninvasive Research Ethics Committee of Fırat University [approval no. 2023/03–15]) according to the Declaration of Helsinki. Written approval was received from Firat University Hospital for the use of the data. The institution requires all patients to provide written consent before surgery, including consent for data use. Data from patients who underwent PD in the same hospital between January 2010 and August 2022 were scanned electronically. Patients aged 18 years and older diagnosed with PAC, evaluated by a multidisciplinary oncology council (medical oncologist, radiologist, and surgical oncologist), who were deemed resectable and underwent PD surgery, were included in the study.

The exclusion criteria were as follows: benign and premalignant pathology; additional organ resection or total pancreatectomy; neoadjuvant therapy; known systemic immune disease; hematologic disease; additional organ cancer; distant organ metastases; chronic liver disease; chronic kidney disease; palliative surgical treatment; and missing data.

Patients included in the study were divided into two groups: Group I included patients who died within 90 days (due to non-cancerous reasons) before being discharged from the hospital or due to reasons requiring rehospitalization after discharge, and Group II included patients who had lived for > 90 days.

### Demographic and laboratory data

Data on age, sex, comorbidities (diabetes mellitus, pulmonary disease, heart disease, and viral hepatitis), age-adjusted Charlson Comorbidity Index (ACCI), American Society of Anesthesiology (ASA), albumin, NLR, CA 19 − 9 levels, MELD-Na scores (at the admission and preoperative), common bile duct diameters, Wirsung canal diameters, and the presence of stents in the common bile duct were recorded. Biochemical values were assessed before biliary drainage (at the first referral). Values for neutrophils, platelets, and lymphocytes, collected in the absence of infection (such as cholangitis) or signs of infection and during periods unrelated to interventional procedures, were included in the study.

### Calculating MELD-Na score

$$\begin{aligned}\mathrm{MELD-Na}&=\mathrm{MELD-Na}\left(\mathrm{mEq/L}\right)\\&-\left(0.025\times\mathrm{MELD}\times\left(140-\mathrm{Na}\right)\right)+140,\end{aligned}$$

$$\begin{aligned}\mathrm{MELD}\left(\mathrm{UNOS}\right)&=\lbrack\lbrack\left(0.957\times\text{Ln Creatinine}\left(\mathrm{mg}/\mathrm{dL}\right)\right)\\&+\left(0.378\times\text{Ln Bilirubin}\left(\mathrm{mg}/\mathrm{dL}\right)\right)\\&+\left(1.12\times\text{Ln INR}\right)\rbrack+0.643\rbrack\times10. \end{aligned}$$

MELD-unos = Model for End-stage Liver Disease - UNOS (score).

(Na + is limited to a range of 125–140).

Entries < 1.0 are set to 1.0 for the purposes of the MELD score calculation.

Ln = natural logarithm.

UNOS = United Network for Organ Sharing.

### Definition of ACCI

The ACCI combines the age equivalence index and the Charlson Comorbidity Index. One point for each additional 10 years of age was added for those over 40 years (e.g.,1 point for those aged 50–59 years and 2 points for those aged 60–69 years). This score was added to the Charlson Comorbidity Index. ACCI was calculated online using MDCalc.

### Surgical procedure

All patients underwent surgery at the same clinic. After a general exploration, the retropancreatic area and portal vein were assessed for resectability. For the surgical margin, resection was planned at the junction of each patient’s superior mesenteric and splenic veins. A frozen examination of the surgical margin was performed. The resection was mostly performed with pylorus resection (PRPD: pylorus-resected PD). Pyloric-sparing PD (PSPD) was performed in a limited number of patients with early-stage disease. Pancreaticojejunostomy was performed as a duct-to-mucosa or dunking technique. Portal vein resection was performed if needed. Surgery was terminated by the routine placement of two drains at the surgical site.

### Peri-postoperative data

Surgical time, perioperative blood loss, pylorus resection, pancreaticojejunostomy anastomosis type, tumor location, portal vein resection, perioperative vasoconstrictor requirement, postoperative sputum culture, and postoperative pancreatic fistula (POPF) were evaluated.

### Assessment of POPF and the study groups

POPF was evaluated and classified according to the International Study Group on Pancreatic Surgery (ISGPS) classification [23]. Drain amylase output, about the length of drain placement, percutaneous or endoscopic interventions, angiographic procedures, reoperation, length of hospital stay, organ failure, and death were assessed for classification. Only grade B and C fistulas were defined and grouped as POPF (+), and grade A was grouped as POPF (-).

### Power analysis

The power analysis was carried out as post-hoc (achieved power) in the WSSPAS (Web-Based Sample Size & Power Analysis Software, URL: http://161.9.167.247/WSSPAS/) because the MELD-Na score has not been previously studied in the literature. The probability of avoiding a type-II error was defined as having a power of more than 80%. The effect size (w) was 0.67, and the power of the study (1-beta) was 0.998 at the alpha error probability of 0.05.

### Statistical analysis

The data were tested for normality of distribution using the Kolmogorov-Smirnov and Shapiro-Wilk tests. Parametric data are expressed as mean ± standard deviation (SD) and nonparametric data as median (minimum-maximum). Parametric data were compared using the independent samples t-test, and nonparametric data were compared using the Mann-Whitney U and Kruskal-Wallis tests. Categorical data were analyzed using the Chi-square or Fisher’s exact test. The repeated measures test was used to compare the repeated measurements between groups. A receiver operating characteristic (ROC) curve determined the optimal cut-off values for prognostic factors. Covariates that showed significant associations with early mortality in univariate analysis (Log rank test) were subjected to multivariate analysis. Multivariate analysis was performed using the Cox proportional hazard regression model. The results are presented as hazard ratios (HR) with 95% confidence intervals (CIs).

## Results

### Patient characteristics

The study included 105 of 148 patients who underwent PD. The mean age of the patients was 62.4 ± 12.2 years, and 67 (63.8%) were male. 17 patients (16.2%) were in Group I, while the others were in group II. Patient demographics and clinical characteristics were presented in Table 1. In cases with 90-day mortality, the male rate and mean age were higher (88.2% vs. 59.1%, *p* = 0.027; 69.9 ± 7.9 years vs. 60.9 ± 12.4 years, *p* = 0.005). There was no difference between the groups in terms of choledochus and Wirsung diameters, ASA scores, comorbidity status (including hepatitis, heart disease, pulmonary disease, and diabetes mellitus), and biliary drainage status (*p* > 0.05 for all). However, there were significant differences in ACCI, albumin levels, NLR, and MELD-Na score upon admission, as well as the preoperative MELD-Na score (*p* < 0.05 for all). There was no difference between the groups in terms of CA 19 − 9 levels (*p* > 0.05).

**Table 1:** Differences of demographic and laboratory data between groups (p values that are less than 0.05 are accentuated in bold)

Variables		Group I (*n* = 17)	Group II (*n* = 88)	*p* value
Age (year)		69.9 ± 7.9	60.9 ± 12.4	**0.005**
Sex	Male	15 (88.2%)	52 (59.1%)	**0.027**
	Female	2 (11.8%)	36 (40.1%)	
ASA score	II	0 (0%)	9 (10.2%)	0.151
	III	15 (88.2%)	75 (85.2%)	
	IV	2 (11.8%)	4 (4.6%)	
ACCI		6 (4–9)	5 (2–9)	**0.002**
Albumin (mg/dL)		3.5 (2.6–4.2)	3.9 (2.8-6)	**0.016**
MELD-Na (Admission)		18.7 ± 5.7	14.9 ± 5.7	**0.013**
MELD-Na (Preoperative)		15.0 ± 5.2	11.6 ± 4.6	**0.007**
NLR		4.6 (1.5–24.8)	2.6 (0.8–46.2)	**0.011**
Neutrophil (10e3/µL)		5.7 (2.5–12.9)	4.6 (2.8–24.9)	0.057
Lymphocyte (10e3/µL)		1.3 (0.2–3.1)	1.6 (0.5–4.9)	0.060
Platelet (10e3/µL)		291 (188–490)	269 (152–647)	0.387
CA 19 − 9 (U/mL)		50 (5.5–3314)	53.6 (0.1-41404)	0.668
Choledochus diameter (mm)		12 (5–25)	14 (4–27)	0.876
Wirsung diameter (mm)		3 (2–12)	4 (2–13)	0.841
Stent in choledochus	Inserted	11 (64.7%)	64 (74.4%)	0.439
	No stent	6 (35.3%)	22 (25.6%)	

*ASA* American Society of Anaesthesiologists score, *NLR* Neutrophil-lymphocyte ratio, *ACCI* Age-adjusted Charlson Comorbidity Index

**Fig. 1:**
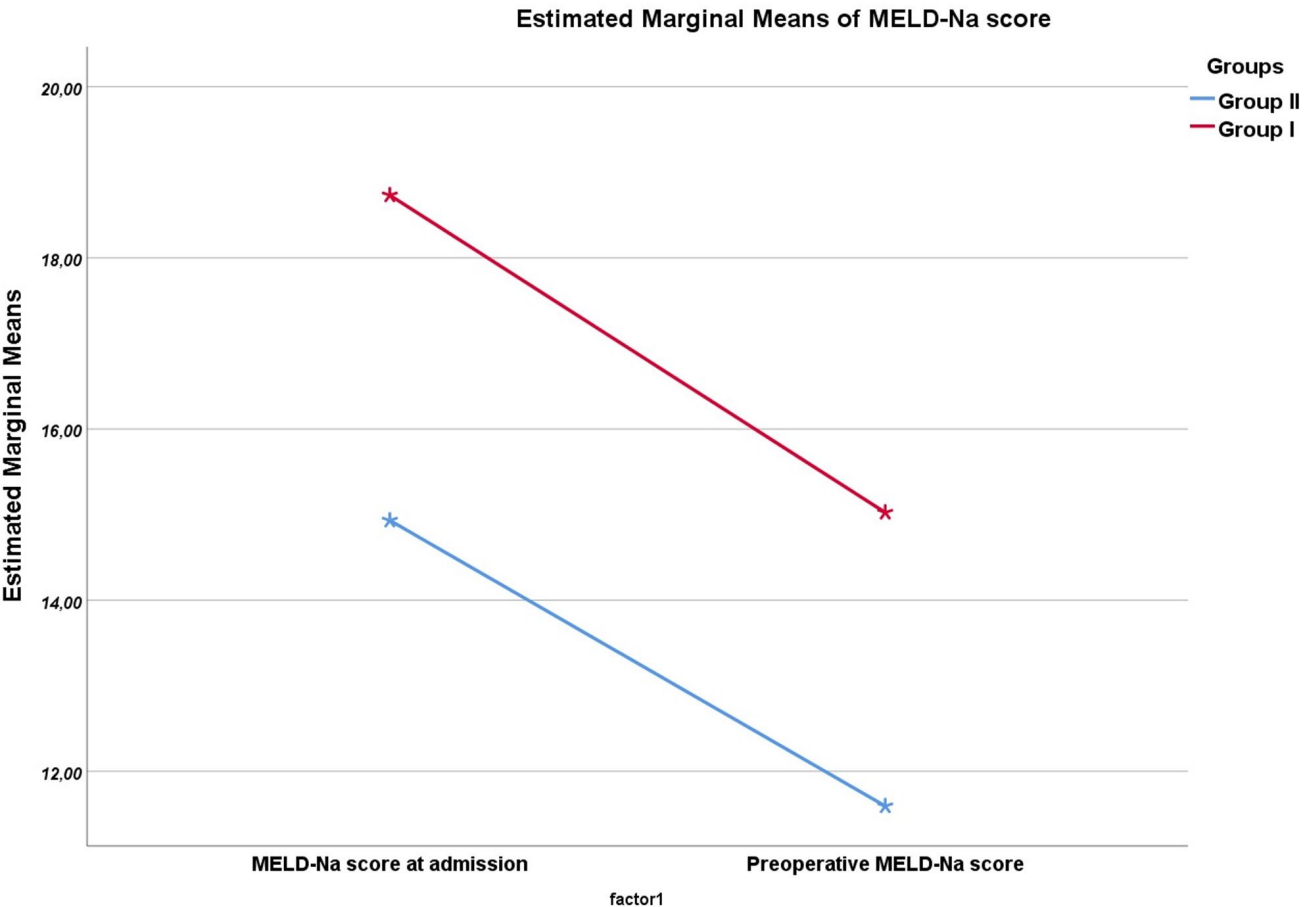
Comparison of the repeated measure of MELD-Na scores of admission and preoperatively between groups (*p* = 0.713)

When the changes between the preoperative and first MELD-Na scores of both groups were compared, no significant difference was observed (*p* = 0.713)(Fig. 1). There was no significant difference between the groups in terms of biliary drainage, and the decrease in MELD-Na scores before and after drainage was similar in both groups.

### Peri-postoperative outcomes

As shown in Table 2, there was a significant difference between gruops in terms of grade B/C POPF (*p* = 0.036) and sputum culture positivity (*p* = 0.002). Blood loss, surgical time, surgery type, anastomosis of the PJ, portal vein resection, vasoconstrictor need, and tumor location were not statistically significantly different between the groups (*p* > 0.05 for all). The causes of death in group I were pneumonia (*n* = 4), bleeding (*n* = 4) [bleeding secondary to POPF (*n* = 2); bleeding from gastrojejunostomy anastomosis (*n* = 2)], pulmonary insufficiency (*n* = 1), sepsis (*n* = 2), cardiovascular collapse (*n* = 2), acute renal failure (*n* = 3), and reoperation due to fistula (*n* = 1).

**Table 2:** Differences of peri-postoperative variables between groups (p values that are less than 0.05 are accentuated in bold)

Variables		Group I (*n* = 17)	Group II (*n* = 88)	*p* value
Operative time (minutes)		365.71 ± 87.65	362.20 ± 62.29	0.844
Surgery type	PRPD	13 (76.5%)	60 (68.2)	0.577
	PSPD	4 (23.5%)	28 (31.8)	
Anastomosis type of PJ	DtM	5 (29.4%)	35 ((39.8%)	0.587
	Dunking	12 (60.6%)	53 (60.2%)	
Portal vein resection	Resected	1 (5.9%)	10 (11.5%)	0.687
	No resection	16 (94.1%)	77 (88.5%)	
Blood loss (ml)		600 (200–1200)	475 (100–1200)	0.170
Vasoconstrictor need	Needed	4 (23.5%)	15 (17.2%)	0.539
	No need	13 (76.5%)	72 (82.8%)	
Tumor location	Pancreatic head	7 (41.2%)	44 (50%)	0.337
	Ampulla	5 (29.4%)	28 (31.8%)	
	Distal choledochus	5 (29.4%)	11 (12.5%)	
	Duodenum	0 (0%)	5 (5.7%)	
POPF	None/Grade A	13 (76.5%)	83 (94.3%)	**0.036**
	Grade B/C	4 (23.5%)	5 (5.7%)	
Postoperative sputum culture	None	11	82	**0.002**
	Klebsiella pneumoniae	2	5	
	Acinetobacter	2	0	
	Pseudomonas	1	1	
	Mixt	1	0	

*DtM* Duct to mucosa, *POPF* Postoperative pancreatic fistula, *PRPD* Pylorus-resected pancreaticoduodenectomy, *PSPD* Pyloric-sparing pancreaticoduodenectomy and *PJ* Pancreaticojejunostomy

### ROC analyses of significant differences between groups

**Fig. 2:**
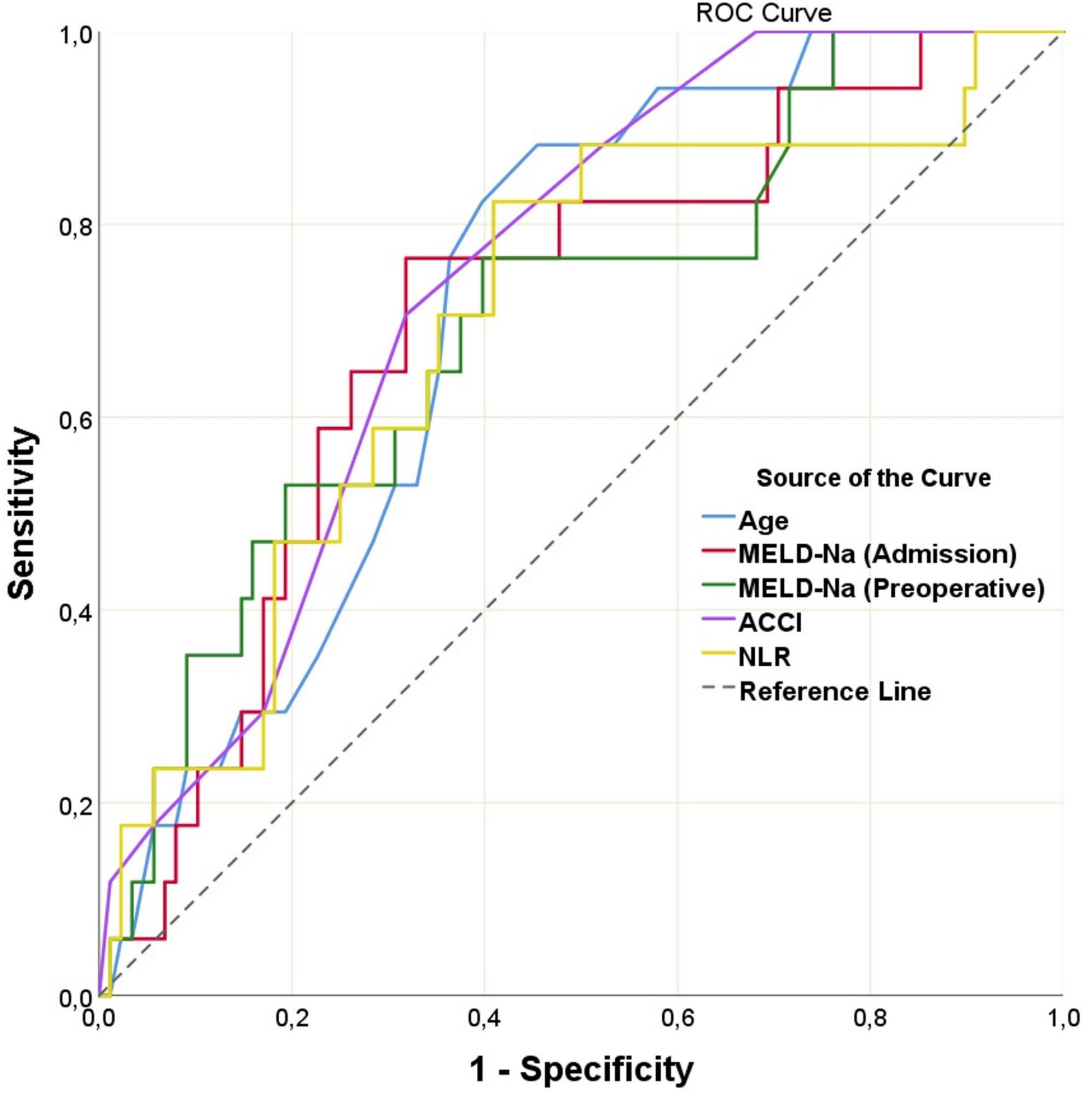
ROC curve analyses of MELD-Na scores, ACCI, Age, and NLR values (AUC values for MELD-Na (Admission): 0.705, MELD-Na (Preoperative): 0.699, ACCI: 0.738, Age: 0.716, and NLR: 0.695 (*p* < 0.05))

Continuous variables significant in the analysis were assessed using ROC analysis. As a result, the most relevant cutoff points with the highest sensitivity and specificity for predicting 90-day mortality were > 16.6 for the MELD-Na score at admission, > 12.3 for the preoperative MELD-Na score, > 5 for the ACCI, ≤ 3.9 mg/dL for albumin, > 3.1 for NLR, and > 63 years for age (Fig. 2).

### Univariate and multivariate analyses for predictors of postoperative 90-day mortality

Univariate and multivariate analyses of the predictive factors related to postoperative 90-day mortality are presented in Table 3. Continuous variables were grouped according to the significant (*p* < 0.05) optimal cut-off values determined in the ROC analysis and included in the Kaplan-Meier (univariate) and Cox regression model (multivariate) analyses. Univariate analysis showed that age (HR: 5.46; 95% CI: [1.94–15.39]; *p* = 0.001), sex (HR: 3.49; 95% CI: [1.19–10.23]; *p* = 0.023), albumin (HR: 3.49; 95% CI: [1.19–10.23]; *p* = 0.023), NLR (HR: 5.19; 95% CI: [1.84–14.59]; *p* = 0.003), ACCI (HR: 5.10; 95% CI: [1.76–14.77]; *p* = 0.003), preoperative MELD-Na score (HR: 4.33; 95% CI: [1.53–12.21]; *p* = 0.006)and MELD-Na score at the admission (HR: 6.41; 95% CI: [2.22–18.50]; *p* < 0.001) were significantly associated with an increased risk of postoperative 90-day mortality. Variables with univariate analysis (*p* < 0.05) were entered into the multivariate Cox proportional hazards model, and the results indicated that the MELD-Na score at the admission (HR: 10.75; 95% CI: [2.82–61.30]; *p* = 0.001), ACCI (HR: 9.30; 95% CI: [2.30-46.05]; *p* < 0.001), and NLR (HR: 7.81; 95% CI: [1.98–48.38]; *p* = 0.005) were independent risk factors for postoperative 90-day mortality. When classified according to cut-off values, the frequency, sensitivity, specificity, PPV, NPV, and accuracy values ​​of the variables were as in Table 4. The MELD-Na score at admission, NLR, and ACCI demonstrated the highest accuracy values.

**Table 3:** Univariate and multivariate analyses of predictive factors of 90-day mortality for PD for PACs (p values that are less than 0.05 are accentuated in bold)

		Univariate analyses			Multivariate analyses		
		HR	95% CI	*p* value	HR	95% CI	*p* value
Age	≤ 63	1.00		**0.001**			
	> 63	5.46	1.94–15.39				
Sex	Female	1.00		**0.023**			
	Male	3.49	1.19–10.23				
Albumin	> 3.9	1.00		**0.023**			
	≤ 3.9	3.49	1.19–10.23				
NLR	≤ 3.1	1.00		**0.003**	1.00		**0.005**
	> 3.1	5.19	1.84–14.59		7.81	1.98–48.38	
MELD-Na score (at admission)	≤ 16.6	1.00		**< 0.001**	1.00		**0.001**
	> 16.6	6.41	2.22–18.50		10.75	2.82–61.30	
MELD-Na score (preoperative)	≤ 12.3	1.00		**0.006**			
	> 12.3	4.33	1.53–12.21				
Stent in choledochus	Inserted	1.00		0.442			
	No stent	0.64	0.20–2.02				
ACCI	≤ 5	1.00		**0.003**	1.00		**< 0.001**
	> 5	5.10	1.76–14.77		9.30	2.30-46.05	

*NLR* Neutrophil-lymphocyte ratio, *ACCI* Age-adjusted Charlson Comorbidity Index

**Table 4:** Frequency, sensitivity, specificity, positive predictive value (PPV), negative predictive value (NPV), and accuracy values ​​of the variables (p values that are less than 0.05 are accentuated in bold)

Variables	Cut-off values	Group I (*n* = 17)	Group II (*n* = 88)	*p* value	Sensitivity -Specifity and PPV-NPV	Accuracy
Age (years)	≤ 63	2 (11.8%)	48 (54.5%)	**0.001**	88.2%-54.6%	60%
	> 63	15 (88.2%)	40 (45.5%)		27.3%-96%	
Albumin (mg/dL)	≤ 3.9	15 (88.2%)	52 (59.1%)	**0.027**	88.2%-40.9%	48.6%
	> 3.9	2 (11.8%)	36 (40.9%)		22.4%-94.7%	
NLR	≤ 3.1	3 (17.6%)	52 (59.1%)	**0.003**	82.4%-59.1%	62.9%
	> 3.1	14 (82.4%)	36 (40.9%)		28%-94.6%	
MELD-Na (Admission)	≤ 16.6	4 (23.5%)	60 (68.2%)	**0.001**	76.5%-68.2%	69.5%
	> 16.6	13 (76.5%)	28 (31.8%)		31.7%-93.8%	
MELD-Na (Preoperative)	≤ 12.3	4 (23.5%)	53 (60.2%)	**0.007**	76.5%-60.2%	62.9%
	> 12.3	13 (76.5%)	35 (39.8%)		27.1%-93%	
ACCI	≤ 5	5 (29.4%)	60 (68.2%)	**0.005**	70.6%-68.3%	68.6%
	> 5	12 (70.6%)	28 (31.8%)		30%-92.3%	
Sex	Male	15 (88.2%)	52 (59.1%)	**0.027**	88.2%-40.9%	48.6%
	Female	2 (11.8%)	36 (40.9%)		22.4%-94.7%	

*NLR* Neutrophil-lymphocyte ratio, *ACCI* Age-adjusted Charlson Comorbidity Index

### Follow up

Except for the 90-day mortality patients, the total follow-up of the patients was 25 ± 21 months for 88 cases. At the start of the study, 29 (33%) patients were alive and 59 (67%) had died, with 33 deaths due to distant metastasis, eight from non-malignant causes, seven from local recurrences, one due to the chemotherapy complication, and ten due to unknown causes.

## Discussion

In this study, the MELD-Na score, ACCI, and NLR were identified as independent risk factors for 90-day mortality in patients undergoing PD for PAC. This is the first study to assess the MELD-Na scores in relation to 90-day mortality for patients undergoing PD for PAC.

The NLR has been investigated in many studies as an indicator of immune function. It has been utilized to assess early mortality in PD and upper gastrointestinal and emergency surgeries for patients with chronic liver disease [20, 21, 24–27]. These studies have shown that NLR is associated with early mortality. Demirelli and Fang reported that the NLR is an independent risk factor for 30-day mortality [25, 26]. The NLR was also found to be an independent risk factor for 90-day mortality in our study. While neutrophils respond to active inflammation, lymphocytes play a regulatory role in the immune pathway. Moreover, neutrophils have the capability to generate reactive oxygen species, nitric oxide, and arginase, leading to the inhibition of the cytotoxic functions of lymphocytes, natural killer cells, and activated T cells [28, 29]. We cannot explain the pathophysiology of the elevated NLR value in group I in our study. However, while there was a difference in the neutrophil and lymphocyte counts between the groups, it was not statistically significant. Neutrophils were high and lymphocytes low in group I, and vice versa in group II. This situation may be secondary to an immune system disorder caused by biliary obstruction [2]. In group I, mortality from pneumonia might be influenced by NLR and endotoxemia.

The comorbidity rate may have increased with age. The ACCI associated with comorbidities was used to estimate the probability of patient survival [30]. ACCI has been studied in pancreatic, gastric, and ampullary cancers and is associated with OS [31, 33]. The ACCI value was high in patients with a short OS. It has also been associated with early mortality. ACCI was not previously investigated for 90-day mortality after PD and was evaluated for the first time in the present study. It was found to be a prognostic risk factor for 90-day mortality. Demirelli, Weger, and Al Abbas reported age as an independent risk factor for PD in their studies [25, 34, 35]. In our study, although age was an independent risk factor in univariate analysis, it was not significant in multivariate analysis. We attribute this to the age effect within the ACCI. Additionally, this difference may have resulted from the fact that comorbidities were evaluated separately in other studies.

Albumin level is a marker of nutritional status in cancer patients. Many patients require preoperative nutritional support to normalize their albumin levels to normal before surgery. Hypoalbuminemia has been shown to be a cause of early (30-day) mortality in patients undergoing PD [25, 27, 36]. In our study, it was also found to be an independent risk factor in univariate analysis for 90-day mortality.

The MELD-Na score, utilized to calculate 90-day mortality in patients with chronic liver disease, is derived from bilirubin, creatinine, INR, and sodium values. Although it is used in chronic liver disease, its ability to predict mortality in many cancer patients has also been studied. Al Abbas et al. found that MELD-Na was significant for 30-day mortality in patients undergoing PD [34]. In their study, Al Abbas used a MELD-Na value of 11 based on the UNOS reference and designed the groups accordingly. Mangieri et al. reported that MELD-Na was not associated with early mortality [37]. The MELD-Na value was also used for postoperative mortality in colon, stomach, and liver cancers, and significant results were also found in these cases [15, 38, 39]. In our study, the MELD-Na score was an independent risk factor for 90-day mortality. In this study, the MELD-Na cut-off value was calculated using ROC analysis and used for the first time for 90-day mortality.

POPF is an important cause of mortality and morbidity in patients undergoing PD [40, 42]. Since this condition developed postoperatively, it was excluded from the regression analysis in our study. However, a statistically significant difference was found between the groups. Two of our patients who developed POPF died from bleeding, and one died from postoperative abdominal sepsis. Reoperation is performed on the 7th postoperative day, and leakage is observed at the posterior wall of the pancreaticojejunostomy anastomosis. The anastomosis was performed again, but the patient died on the 14th postoperative day from abdominal sepsis.

In PACs, bilirubin and bile acid excretion into the intestine may be partially or entirely obstructed secondary to biliary obstruction. This may adversely affect the liver, kidneys, immune system, hemostasis, wound healing, and other systems [2]. Studies on the causes of early postoperative death in PACs, mostly sepsis, bleeding, and organ failure, are at the forefront [43].

Biliary obstruction leads to the stasis of bile acids, which are involved in glucose metabolism, obesity, thyroid function, the cardiovascular system, renal function, and numerous other physiological processes [44]. Bile acids can also influence the cardiovascular system by binding to some nuclear and membrane receptors, and Ca^+^-activating K^+^ channels. Bile acids damage cardiomyocytes and vascular cells, causing bradycardia and decreased systemic vascular resistance. This suggests that biliary obstruction may cause early mortality via the cardiovascular system. In our study, two patients died from cardiovascular collapse.

Biliary obstruction can cause acute kidney damage or renal failure due to the accumulation of bile acids in renal tubules when reabsorption is exceeded [45]. This mechanism of damage leads to increased creatinine levels in patients. Many studies have emphasized that creatinine is an independent risk factor for postoperative mortality [36, 46, 47]. The mortality rate for patients undergoing surgery for this condition and developing renal failure is high, at 70% to 80%^2^. In our study, three patients died from postoperative renal failure.

Hyperbilirubinemia increases the tendency to bleed by decreasing platelet aggregation [48]. This may cause postoperative gastrointestinal bleeding or pancreatic hemorrhage. Studies have found that perioperative and postoperative hemorrhages are important causes of morbidity and mortality [43]. In our study, four patients died of postoperative hemorrhage. A patient with GI bleeding developed sudden hypotension postoperatively and was admitted to the ICU. The patient died prior to the surgery. In the other patient, low hemoglobin levels were detected, and a transfusion was administered. During follow-up, the patient’s bleeding was ceased with two endoscopic procedures, and a CT angiogram was performed, revealing no active bleeding source. This patient was also transferred to the ICU after a sudden drop in hemoglobin but died. One of the two patients with bleeding caused by the fistula was arrested in the ICU after sudden hypotension during follow-up and died. The other patient’s bleeding was monitored, and a percutaneous drainage was performed. This patient also died following sudden hypotension. Bilirubin may inhibit platelet activation or aggregation via; scavenging H2O2 to block arachidonic acid mobilization, blocking collagen receptor function, inhibiting the production of platelet derived thromboplastin and platelet factor 3, preventing complement mediated platelet activation by inhibiting several complement proteins, and thrombus formation by inhibiting P-selectin release [48]. Investigating platelet function in patients undergoing PD may be helpful in preventing postoperative hemorrhage.

Endotoxemia can occur when bile acids do not enter the intestines, weakening the intestinal barrier and permitting endotoxins into systemic circulation, which may cause immune disorders [2]. Hyperbilirubinemia caused by biliary obstruction has been reported to lead to immune dysfunction through NF-kB, tumor necrosis factor-α (TNF-α), and TLR4 [49, 50]. It is also known that TNF-α causes hepatotoxicity [2]. These mechanisms and other effects may lead to immune dysfunction, resulting in infection-related mortality in PACs [51]. In our study, four patients died from pneumonia, and two patients died from sepsis. While the blood cultures of two patients who developed sepsis tested positive, the urine, sputum, and wound cultures were negative. Abdominal imaging revealed no signs of collection or leakage. Both patients’ NLR and ACCI values, as well as one patient’s MELD-Na values, were above the cut-off level. Both patients died due to multiple organ failure secondary to sepsis. Sputum cultures from 3 of 4 patients who developed pneumonia were monobacterial, while one was polybacterial. One patient who died from pneumonia had POPF, but this fistula was under control. One patient underwent two postoperative laparotomies and also died of pneumonia. All four patients died from sepsis and multiple organ failure caused by pneumonia. The literature indicates that both pneumonia and sepsis are common causes of postoperative mortality after PD [52].

Our high mortality rate may be due to the hospital volume and surgeons’ experience [7–10]. Not all patients were operated on by a hepatopancreatobiliary surgeon. Moreover, in the recent study, it is reported as 17.8% and 24.3% [53]. This could be related to the hospital experience and the lack of a hepatopancreatobiliary surgeon or poor patient management in the intensive care unit.

In the study, we used the laboratory results before the biliary drainage. The reason for this, although controversial, was the ineffectiveness of biliary drainage on the mortality and morbidity rates reported in the literature [53–56]. The NCCN guideline does not recommend routine drainage in these patient groups [57]. Furthermore, many studies and meta-analyses have found no association between drainage and mortality [53–55, 58–60]. Although it has been reported that a 2-week period is required for recovery of hepatocyte functions, its relationship with morbidity has not been reported [54]. Additionally, no difference was reported in terms of multidrug-resistant bacterial colonization [55].

High MELD-Na score, ACCI, and NLR in these patients are considered risk factors for mortality. The main goal is to alter changeable situations and take precautions. Just as a patient with pulmonary disease performing breathing exercises, a multidisciplinary team evaluation is important to minimize problems involving the renal, cardiovascular, and other systems. Additionally, as mentioned above, it is essential to evaluate platelet function before surgery or to assess the patient for biliary drainage. Assessing patients with high NLR for the risk of developing sepsis or pneumonia and reviewing them for antibiotic prophylaxis may reduce mortality.

Our study had some limitations. The retrospective nature of the study at a single center, the limited number of patients, the low annual case volume, and the high mortality rate compared to the literature may have influenced the study results. The finding that the preoperative MELD-Na score was insignificant in multivariate analysis may be related to the multicollinearity with the MELD-Na score at admission.

## Conclusion

The MELD-Na score, ACCI, and NLR were independent risk factors for 90-day mortality following PD. The MELD-Na score at admission had a greater impact than the preoperative MELD-Na score. Evaluating patients in high-risk groups with a multidisciplinary approach may reduce mortality rates. Further prospective high-volume studies are needed for more reliable results.

## Data Availability

The data supporting this study’s findings will be available from the corresponding author upon reasonable request.

## Abbreviations

PD: Pancreaticoduodenectomy
ACCI: The age-adjusted Charlson Comorbidity Index
NLR: Neutrophil-lymphocyte ratio
PAC: Periampullary cancers
OS: Overall survival
MELD: Model for End-Stage Liver Disease
ASA: American Society of Anesthesiology
PRPD: Pylorus-resected PD
PSPD: Pyloric-sparing PD
POPF: Postoperative pancreatic fistula
ROC: Receiver operating characteristic
HR: Hazard ratios
PPV: Positive predictive value
NPV: Negative predictive value
